# Target Tracking in Clutter with Extremum Seeking Control for Adaptive Detection Thresholding

**DOI:** 10.3390/s19061386

**Published:** 2019-03-20

**Authors:** Seung Hyo Park, Taek Lyul Song

**Affiliations:** Department of Electronic Systems Engineering, Hanyang University, Ansan 15588, Korea; gyeonwoo4@naver.com

**Keywords:** target tracking, probability density function of signal strength, detection threshold, estimated clutter measurement density, desired clutter measurement density, extremum seeking control

## Abstract

If the signal strength obtained from sonar is higher than the predefined detection threshold, it is considered as a candidate for target tracking. This detection threshold is a parameter that affects the detection probability of targets and the distribution of clutter measurements, so it is important to determine a proper threshold to improve target tracking performance. There are various techniques for adjusting the detection threshold. To apply these techniques, it is assumed that the probability density functions of the signal strength for clutter are known in advance. However, in a real environment, the probability density function of the signal strength is unknown in general. In this paper, we propose a detection threshold control method using extremum seeking control in realistic environments. The extremum seeking control is a method used in complex nonlinear systems. We propose a new structure for extremum seeking control that is applicable to detection processes with nonlinear characteristics. This structure is used to adjust the detection threshold of the received signal amplitude to make the estimated clutter measurement density converge to a designed clutter measurement density to achieve the best target tracking performance in the current environment. Simulation studies for the proposed extremum seeking control applied to target tracking in an unknown clutter signal distribution demonstrate the effectiveness and improved target tracking performance.

## 1. Introduction

Signals acquired from sonar are used as measurements for track initiation and track updating if the signal amplitude is higher than the specified detection threshold. The clutter refers to the non-target random detections generated by random objects, thermal noise effects, reverberation, etc., that are generally random in number, location, and intensity. The sonar acquires the clutter measurements in addition to the target measurements, so that the target tracking performance is deteriorated by these clutter measurements. Therefore, to improve the target tracking performance in a cluttered environment, data association [1,2,3,4] that distinguishes the target from clutter is essential. The number of clutter measurements in a surveillance region is usually modeled by a Poisson process, which is parameterized by the clutter measurement density. The clutter measurement density represents the average number of the clutter measurements per unit volume (area) of the surveillance region. The clutter measurement density is an important parameter used in most of the algorithms for target tracking in clutter for calculating the data association probability of each measurement, which provides a probabilistic weight for target state update. In most data association techniques, it is assumed that the spatial distribution of clutter measurement is “homogeneous” and the clutter measurement density is known in advance. Since the spatial distribution of clutter measurements is unknown in a real environment, various clutter measurement density estimation techniques have been proposed [5,6]. The estimated clutter measurement density value of these measurements affects target tracking performance, and the clutter measurement density is a parameter that can be changed via the detection threshold. Therefore, it is desirable to set the detection threshold that provides the clutter measurement density for achieving the maximum target tracking performance for the environment.

However, there is trade-off in varying the detection threshold. If the detection threshold is increased, the number of generated clutter measurements is small, and the clutter measurement density is low; however, the detection probability of targets is also low. Conversely, lowering the detection threshold increases the detection probability of the target, but the clutter measurement density increases [7].

Techniques such as “CFAR” for determining an appropriate detection threshold in the presence of such trade-offs have been proposed [8,9]. However, in order to use “CFAR”, it is necessary to assume that the probability distribution of the signal strength for clutter is known in advance. Since the prior information of the signal strength of clutter cannot be known, a technique capable of adjusting the detection threshold is required in real tracking environments.

In this paper, we propose a detection threshold control method applying extremum seeking control, assuming that the probability density function of the signal strength for clutter is unknown [10]. The target used in this study is a surface ship moving on shallow water, and an active sonar positioned underwater near the surface of the sea is employed to track the target as a part of a harbor defense system. The target movement is confined in a two-dimensional space where the clutter measurements due to reverberations can be densely populated. Extremum seeking control is an optimization approach to dynamic problems using limited information in a nonlinear system that knows only preliminary information, that it has a minimum or maximum extremum [11,12,13,14,15]. This extremum seeking control is also used in ABS (anti-lock braking system) [16], WECS (wind energy conversion system) [17], and VCS (vapor compression system) [18]. In this way, extremum seeking control has recently been widely utilized in various fields with nonlinear systems. Since the detection process in a real environment has nonlinear characteristics and the clutter signal appears at the tail of the clutter signal strength probability density function, the clutter measurement density can vary significantly even with small changes in the detection threshold. That is, a small change in the detection threshold has a significant effect on the target tracking performance. To perform adaptive detection threshold control under these difficult conditions, we designed a new extremum seeking control structure that can be applied in the detection process. The extremum seeking control of the new structure compares the average value of the clutter measurement density of the measurements at the current scan with the desired clutter measurement density value. Then, it calculates the detection threshold of the next scan as an output so that the average value of the clutter measurement density of the measurements of the next scan converges to the desired clutter measurement density value. The desired clutter measurement density value is selected from the optimum clutter measurement density value to achieve the maximum target tracking performance [7] while varying the detection probability of the target for a given SNR.

The clutter measurement density is calculated by estimating the specific spatial volume for each measurement through SCMDE (spatial clutter measurement density estimation) proposed in [6].

In order to verify the effectiveness of the proposed method, a series of simulation runs for target tracking with unknown clutter distribution information is executed. The simulation scenarios include constant velocity motions in homogeneous clutter environments and a maneuvering motion in a heterogeneous clutter environment. To improve the tracking performance, the interacting multiple model (IMM) [19] structure with several possible target dynamic models is employed for maneuvering target tracking in the simulation together with the proposed extremum seeking control, which controls the signal strength by setting a threshold to match the current spatial clutter density to the desired spatial clutter density. As automatic track initialization leads to the false tracks from the clutter measurements in addition to the true tracks, false track discrimination (FTD) is required to eliminate the false tracks. The integrated probabilistic data association (IPDA) algorithm [20], which utilizes the target existence probability as a track quality measure, is also applied in this paper for discriminating the false tracks.

The rest of this paper is organized as follows. The problem statement for modeling and target tracking algorithm is presented in Section 2. Section 3 describes the basic theory of extremum seeking control and the proposed extremum seeking control structure for adjusting the detection threshold. Section 4 explains the algorithm to find the desired clutter measurement density, which is the input of extremum seeking control. Target tracking performance improvement of the proposed algorithm through the extremum seeking control is analyzed from performance comparison of the proposed algorithm and the tracking filter using the fixed threshold, which is commonly used in practice by Monte Carlo simulation in Section 5, followed by concluding remarks in Section 6.

## 2. Problem Statement

A target tracking algorithm based on the Kalman filter is used to estimate the state variable of the target being tracked. To use this algorithm, it is necessary to design the measurement model and kinetic model of the target to be tracked mathematically. In addition, since the measurement information obtained from the sonar includes not only the target measurements, but also the clutter measurements (unwanted information), a clutter measurement distribution model is needed. To perform target tracking in the presence of clutter, it is necessary to use data association to distinguish between the target measurements and the clutter measurements. However, target tracking algorithms based on data association do not have a means to judge whether the track is tracking the target or clutter. To overcome these problems, data association target tracking algorithms based on track management have been proposed. Track management is a technique for assigning the concept of “target existence probability”, which is a means of evaluating tracks, to each track and determining whether to perform target tracking based on the target existence probability. In this section, the target dynamics, measurements obtained from the sensor, clutter measurement model, and IMM-IPDA [21] are described.

### 2.1. Target Dynamics

The target for this study is a surface ship moving on shallow water such that the target state dynamic equation is modeled in a two-dimensional Cartesian coordinate system with the target state $x_{k}$ satisfying:

(1) $$\begin{array}{c} x_{k}=\left[x,y,\dot{x},\dot{y}\right], \end{array}$$

where *x* and *y* are the position components and $\dot{x}$ and $\dot{y}$ are the velocity components. The dynamic model of the target is equal to:

(2) $$\begin{array}{c} x_{k+1}=F_{k}x_{k}+w_{k} \end{array}$$

where $F_{k}$ is the state transition matrix and $w_{k}$ is the white Gaussian process noise with zero mean and covariance of $Q_{k}$.

For maneuvering target dynamics, the state transition matrix F can be described by either $F_{k}^{1}$ and $F_{k}^{2}$ where $F_{k}^{1}$ is used for constant velocity (CV) dynamics, whereas $F_{k}^{2}$ is used for constant turn rate (CTR) dynamics, respectively [22].

(3) $$\begin{array}{c} F_{k}^{1}=\left[\begin{array}{cc} I_{2} & \Delta I_{2}\\ 0_{2} & I_{2} \end{array}\right]\;\; \end{array}$$

(4) $$\begin{array}{c} F_{k}^{2}=\left[\begin{array}{cccc} 1 & 0 & \frac{\sin\left(\Omega \Delta \right)}{\Omega } & \frac{-(1-\cos\left(\Omega \Delta \right))}{\Omega }\\ 0 & 1 & \frac{1-\cos\left(\Omega \Delta \right)}{\Omega } & \frac{\sin\left(\Omega \Delta \right)}{\Omega }\\ 0 & 0 & \cos\left(\Omega \Delta \right) & -\sin\left(\Omega \Delta \right)\\ 0 & 0 & \sin\left(\Omega \Delta \right) & \cos\left(\Omega \Delta \right) \end{array}\right] \end{array}$$

Here, $I_{n}$ and $0_{n}$ denote the n×n identity and zero matrices, respectively, and Δ and Ω are the interval and the target turn rate. The positive sign of Ω represents the CW (clockwise) turn of the target, and the negative sign of Ω represents the CCW (counter-clockwise) turn, respectively.

### 2.2. Measurement

The active sonar measures both the range $y_{k}^{r}$ and bearing $y_{k}^{\theta }$ of the target. The measurement equation is given by:

(5) $$\begin{array}{c} y_{k}=h{\left(x\right)}_{k}+v_{k} \end{array}$$

where $v_{k}$ is the white Gaussian measurement noise with zero mean and covariance of $W_{k}$.

(6) $$\begin{array}{c} h{\left(x\right)}_{k}=\left[\begin{array}{c} y_{k}^{r}\\ y_{k}^{\theta } \end{array}\right]\;\;=\left[\begin{array}{c} \sqrt{{\left(x-x^{sonar}\right)}^{2}+{\left(y-y^{sonar}\right)}^{2}}\\ \tan^{-1}\left(\frac{y-y^{sonar}}{x-x^{sonar}}\right) \end{array}\right]\;\; \end{array}$$

(7) $$\begin{array}{c} W_{k}=\left[\begin{array}{cc} \sigma_{k,r}^{2} & 0\\ 0 & \sigma_{k,\theta }^{2} \end{array}\right]\;\; \end{array}$$

Here, $x^{sonar}$ and $y^{sonar}$ denote the position in Cartesian coordinates.

In this paper, the conversion method [23] that converts the measurements $y_{k}$ in polar coordinates to the measurement $z_{k}$ in Cartesian coordinates is used. The covariance matrix of the converted measurement $R_{k}$ can be expressed by using the Jacobian matrix $J_{k}$.

(8) $$\begin{array}{c} z_{k}=\left[\begin{array}{c} z_{k}^{x}\\ z_{k}^{y} \end{array}\right]\;\;=\left[\begin{array}{c} y_{k}^{r}\cos\left(y_{k}^{\theta }\right)\\ y_{k}^{r}\sin\left(y_{k}^{\theta }\right) \end{array}\right]\;\; \end{array}$$

(9) $$\begin{array}{c} R_{k}=J_{k}W_{k}J_{k}^{T} \end{array}$$

where:

(10) $$\begin{array}{c} J_{k}=\left[\begin{array}{cc} \frac{\partial z_{k}^{x}}{\partial y_{k}^{r}} & \frac{\partial z_{k}^{x}}{\partial y_{k}^{\theta }}\\ \frac{\partial z_{k}^{y}}{\partial y_{k}^{r}} & \frac{\partial z_{k}^{y}}{\partial y_{k}^{\theta }} \end{array}\right]\;\;=\left[\begin{array}{cc} \cos y_{k}^{\theta } & -y_{k}^{r}\sin y_{k}^{\theta }\\ \sin y_{k}^{\theta } & y_{k}^{r}\cos y_{k}^{\theta } \end{array}\right]\;\; \end{array}$$

$z_{k}$ represents the measurements obtained by the active sonar at the current scan *k*. A collection of all measurements up to time *k* can be expressed as the measurement set $Z^{k}=\left\{Z^{k-1}\cup z_{k}\right\}$.

### 2.3. Clutter

The number of clutter measurements in the surveillance region follows the Poisson distribution. It is expressed as follows.

(11) $$\begin{array}{c} P\left(m\right)=\mu \left(m,\hat{m}\right)=\frac{{\left(\hat{m}\right)}^{m}}{m!}e^{-\hat{m}} \end{array}$$

where $\hat{m}$ represents the average number of clutter measurements in the surveillance region and *m* represents the number of clutter measurements obtained from the sensor in the surveillance region. Since the distribution of clutter measurements in the surveillance region is non-homogeneous, the clutter measurement density is calculated by estimating the clutter measurement density as an unknown parameter.

The probability distribution of the clutter signal strength is assumed to follow a Chi-squared, Rayleigh, or Weibull distribution in most tracking systems, and each probability distribution is respectively expressed below [24].

(12) $$\begin{array}{c} P_{s}^{Chi}\left(s\right)=e^{-s} \end{array}$$

(13) $$\begin{array}{c} P_{s}^{Ray}\left(s\right)=se^{\frac{-s^{2}}{2}} \end{array}$$

(14) $$\begin{array}{c} P_{s}^{Wei}\left(s\right)=\frac{c}{b}{\left(\frac{s}{b}\right)}^{c-1}e^{{\left(\frac{-s}{b}\right)}^{c}} \end{array}$$

Here, *b* is a scale parameter, and cis a shape parameter.

In this paper, the probability distribution of clutter signal strength is modeled as “exponential-type” with unknown parameter λ as:

(15) $$\begin{array}{c} P_{s}\left(s\right)=\lambda e^{-\lambda s} \end{array}$$

For the exponential distribution, 1/λ is the mean and $1/\lambda^{2}$ is the variance of the signal strength. If λ gets smaller, the distribution has a heavier tail. If λ=1, it is the same as the Chi-squared distribution. In actual tracking environments, however, this λ may have a value other than one. When the probability distribution of the clutter signal strength is equal to (15), the probability of false alarm can be expressed as follows.

(16) $$\begin{array}{c} P_{fa}=\int_{\tau }^{\infty }P_{s}\left(s\right)ds \end{array}$$

where τ is the detection threshold for the signal strength.

Since the probability distribution of clutter signal strength modeled in exponential form has a large tail, the probability of false alarm can change drastically even with small changes in the detection threshold.

### 2.4. IMM-IPDA Algorithm Description

Data association techniques such as IPDA are essential because the measurements obtained by the active sonar involve both clutter measurements and target measurements. The IPDA algorithm utilizes the target existence probability as a track quality measure. The target existence probability is calculated recursively based on the received measurements at each scan. The track management of IPDA determines confirmation and termination of the tracks based on the updated target existence probability. The presence of false tracks constrains the application of the IMM algorithm for tracking targets in cluttered environments such that the IMM-IPDA algorithm is proposed. The IMM-IPDA algorithm consists of an interaction step, a prediction step, a validated measurement selection step, an update step, and a combination step, inside a single sampling time.

In the interaction step, the predicted mode probability is calculated by using the mode transition probability $\pi_{\left(m,n\right)}$ and the updated mode probability of each dynamic model. The dynamic models at the time k−1 and *k* are indexed by *n* and *m*, respectively. The mode change between consecutive scans is assumed to follow the Markov process. The mixed state vector ${\tilde{x}}_{k-1|k-1}^{m|n}$ and the error covariance matrix ${\tilde{P}}_{k-1|k-1}^{m|n}$ are calculated by:

(17) $$\begin{array}{c} {\tilde{x}}_{k-1|k-1}^{m|n}=\sum_{n=1}^{N}\mu_{k-1|k-1}^{m|n}{\hat{x}}_{k-1|k-1}^{n} \end{array}$$

(18) $$\begin{array}{c} {\tilde{P}}_{k-1|k-1}^{m|n}=\mu_{k-1|k-1}^{m|n}\sum_{n=1}^{N}\left[\left(P_{k-1|k-1}^{n}+{\hat{x}}_{k-1|k-1}^{n}{\left({\hat{x}}_{k-1|k-1}^{n}\right)}^{T}\right)\right]-{\tilde{x}}_{k-1|k-1}^{m|n}{\left({\tilde{x}}_{k-1|k-1}^{m|n}\right)}^{T} \end{array}$$

where $\mu_{k-1|k-1}^{m|n}$ is the mixing probability given by:

(19) $$\begin{array}{c} \mu_{k-1|k-1}^{m|n}=\frac{\pi_{\left(m,n\right)}\mu_{k-1|k-1}^{n}}{\mu_{k|k-1}^{m}} \end{array}$$

The predicted mode probability is:

(20) $$\begin{array}{c} \mu_{k|k-1}^{m}=\sum_{n=1}^{N}\pi_{\left(m,n\right)}\mu_{k-1|k-1}^{n} \end{array}$$

where *N* is the dynamic model number, and $\pi_{\left(m,n\right)}$ is the transition probability given by:

(21) $$\begin{array}{c} \pi_{\left(m,n\right)}=\left[\begin{array}{ccc} 0.98 & 0.01 & 0.01\\ 0.01 & 0.98 & 0.01\\ 0.01 & 0.01 & 0.98 \end{array}\right] \end{array}$$

The diagonal elements represent the probability that the dynamics model of the previous time is the same dynamic model at the next time.

In the prediction step, a Kalman filter prediction step is performed for each dynamic model.

(22) $$\begin{array}{c} {\hat{x}}_{k|k-1}^{m}=F_{k}^{m}{\tilde{x}}_{k-1|k-1}^{m} \end{array}$$

(23) $$\begin{array}{c} P_{k|k-1}^{m}=F_{k}^{m}{\tilde{P}}_{k-1|k-1}^{m}{\left(F_{k}^{m}\right)}^{T}+Q_{k}^{m} \end{array}$$

The prior target existence probability $P\left\{\chi_{k}|Z^{k-1}\right\}$ is modeled as Markov Chain 1 and is calculated by the following equation.

(24) $$\begin{array}{c} P\left\{\chi_{k}|Z^{k-1}\right\}=P\left\{\chi_{k}|\chi_{k-1}\right\}P\left\{\chi_{k-1}|Z^{k-1}\right\}+P\left\{\chi_{k}|{\bar{\chi }}_{k-1}\right\}P\left\{{\bar{\chi }}_{k-1}|Z^{k-1}\right\} \end{array}$$

Here, $P\left\{\chi_{k}|\chi_{k-1}\right\}$ is the probability of the event in which the target exists both at time k−1 and *k*, and $P\left\{\chi_{k}|{\bar{\chi }}_{k-1}\right\}$ is the probability of the event in which the target does not exist at time k−1 and the target exists at the time *k*.

In the validated measurement selection step, the validation gate is set around the predicted state vector of each dynamic model, and then, the measurements in this area are used for track state update. Measurement $z_{k,i}$ becomes a validated measurement when it satisfies the condition of:

(25) $$\begin{array}{c} {\left(z_{k,i}-H{\hat{x}}_{k|k-1}^{m}\right)}^{T}{\left(S_{k,i}^{m}\right)}^{-1}\left(z_{k,i}-H{\hat{x}}_{k|k-1}^{m}\right)<\gamma_{G} \end{array}$$

where $\gamma_{G}$ is the validation gate size, and the measurement matrix H and the predicted measurement error covariance matrix $S_{k,i}^{m}$ are given by:

(26) $$\begin{array}{c} S_{k,i}^{m}=HP_{k|k-1}^{m}H^{T}+R_{k,i}. \end{array}$$

(27) $$\begin{array}{c} H=\left[\begin{array}{cc} I_{2} & 0_{2} \end{array}\right]\;\; \end{array}$$

The likelihood for the validated measurement of the $m^{\mathrm{th}}$ dynamic model $p_{k,i}^{m}$ is given by the following equation, and the likelihood for the track $p_{k,i}$ can be calculated as follows.

(28) $$\begin{array}{c} p_{k,i}^{m}=\frac{N(z_{k,i};H{\hat{x}}_{k|k-1}^{m},S_{k,i}^{m})}{P_{G}} \end{array}$$

(29) $$\begin{array}{c} p_{k,i}=\sum_{m=1}^{N}\mu_{k|k-1}^{m}p_{k,i}^{m} \end{array}$$

where $P_{G}$ is defined as the probability that a measurement is within the validation gate.

In the update step, the posterior mode probability of each dynamic model $\mu_{k|k}^{m}$ is expressed by the following equation.

(30) $$\begin{array}{c} \mu_{k|k}^{m}=\sum_{i=0}^{M_{k}^{m}}\mu_{k|k,i}^{m}\beta_{k,i} \end{array}$$

where $M_{k}^{m}$ is denoted by the number of validated measurements for the *m*th dynamic model and:

(31) $$\begin{array}{c} \mu_{k|k,i}^{m}=\left\{\begin{array}{cc} \mu_{k|k-1}^{m}\frac{p_{k,i}^{m}}{p_{k,i}} & i>0\\ \mu_{k|k-1}^{m} & i=0 \end{array}\right. \end{array}$$

Data association probability $\beta_{k,i}$ indicates the probability that measurement $z_{k,i}$ originates from the target and is calculated by:

(32) $$\begin{array}{c} \beta_{k,i}=\left\{\begin{array}{cc} \frac{P_{D}P_{G}}{\Lambda_{k}}\frac{p_{k,i}}{\rho_{k,i}} & i>0\\ \frac{1-P_{D}P_{G}}{\Lambda_{k}} & i=0 \end{array}\right. \end{array}$$

The measurement likelihood ratio $\Lambda_{k}$ is:

(33) $$\begin{array}{c} \Lambda_{k}=1-P_{D}P_{G}+P_{D}P_{G}\sum_{i=1}^{M_{k}}\frac{p_{k,i}^{\tau ,p}}{\rho_{k,i}} \end{array}$$

where $M_{k}$ is the number of validated measurements for the track and $P_{D}$ and $\rho_{k,i}$ are the target detection probability and the clutter measurement density at spatial point $z_{k,i}$, respectively. The data association probability $\beta_{k,i}$ in (32) is expressed as the ratio of likelihood $p_{k,i}$ and clutter measurement density $\rho_{k,i}$, which implies that the clutter measurement density impacts the tracking performance significantly.

The data association probability for each dynamic model $\beta_{k,i}^{m}$ is:

(34) $$\begin{array}{c} \beta_{k,i}^{m}=\beta_{k,i}\frac{\mu_{k|k,i}^{m}}{\mu_{k|k}^{m}} \end{array}$$

The posterior state vector ${\hat{x}}_{k|k}^{m}$ and the posterior error covariance matrix $P_{k|k}^{m}$ can be calculated through a Gaussian mixture using the data association probability for each dynamic model $\beta_{k,i}^{m}$ as a weight.

(35) $$\begin{array}{c} {\hat{x}}_{k|k}^{m}=\sum_{i=0}^{M_{k}^{m}}\beta_{k,i}^{m}{\hat{x}}_{k|k,i}^{m} \end{array}$$

(36) $$\begin{array}{c} P_{k|k}^{m}=\sum_{i=0}^{M_{k}^{m}}\beta_{k,i}^{m}\left(P_{k|k,i}^{m}+{\left({\hat{x}}_{k|k,i}^{m}{\hat{x}}_{k|k,i}^{m}\right)}^{T}\right)-{\hat{x}}_{k|k}^{m}{\left({\hat{x}}_{k|k}^{m}\right)}^{T} \end{array}$$

where,

(37) $$\begin{array}{c} {\hat{x}}_{k|k,i}^{m}={\hat{x}}_{k|k-1}^{m}+K_{k,i}^{m}\left(z_{k,i}-H{\hat{x}}_{k|k-1}^{m}\right) \end{array}$$

(38) $$\begin{array}{c} P_{k|k,i}^{m}=\left(I-K_{k,i}^{m}H\right)P_{k|k-1}^{m} \end{array}$$

(39) $$\begin{array}{c} K_{k}^{m}=P_{k|k-1}^{m}{\left(H^{m}\right)}^{T}{\left(S_{k,i}^{m}\right)}^{-1} \end{array}$$

The posterior target existence probability is calculated using the measurement likelihood ratio $\Lambda_{k}$ such that:

(40) $$\begin{array}{c} P\left\{\chi_{k}|Z^{k}\right\}=\frac{P\left\{\chi_{k}|Z^{k-1}\right\}\Lambda_{k}}{1-\left(1-\Lambda_{k}\right)P\left\{\chi_{k}|Z^{k-1}\right\}} \end{array}$$

In the combination stage, the final output of the track is calculated. The estimate of the track can be obtained from the Gaussian mixture for all dynamic models using the mode probability of the dynamic model as a weight.

(41) $$\begin{array}{c} {\hat{x}}_{k|k}=\sum_{m=1}^{N}\mu_{k|k}^{m}{\hat{x}}_{k|k}^{m} \end{array}$$

(42) $$\begin{array}{c} P_{k|k}=\sum_{m=1}^{N}\mu_{k|k}^{m}\left(P_{k|k}^{m}+{\left({\hat{x}}_{k|k}^{m}{\hat{x}}_{k|k}^{m}\right)}^{T}\right)-{\hat{x}}_{k|k}{\left({\hat{x}}_{k|k}\right)}^{T} \end{array}$$

## 3. Extremum Seeking Control for Controlling the Detection Threshold

**Figure 1:**
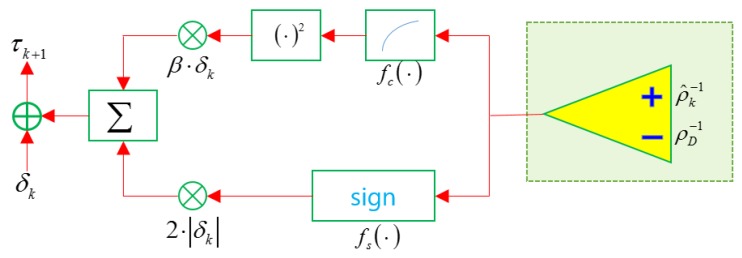
Extremum seeking control structure for adjusting the detection threshold.

The goal is to adjust the detection threshold so that the estimated clutter measurement density $\hat{\rho_{k}}$ for the measurements converges to the desired clutter measurement density $\rho_{D}$. In other words, the input is the inverse of the clutter measurement density, and the output is the detection threshold; thus, it cannot be implemented with the existing extremum seeking control structure. In this section, we propose an extremum seeking control structure considering the nonlinearity between the input and output values. Considering the exponential signal amplitude probability distribution of clutter, as shown in (16), the transfer function is characterized by a non-increasing function. This means that there is no local extreme value and that it has only one extremum after applying the square function. In this case, extremum seeking control guarantees convergence to the global extremum [10,13]. The block diagram is shown in Figure 1. The extremum seeking control structure in this subsection is divided into two parts considering the detection process. The upper part serves to find the extremum, and the lower part plays the role of determining the sign.

There are two input values. The inverse of the estimated clutter measurement density ${\hat{\rho }}_{k}^{-1}$ enters the plus input, and the inverse of the desired clutter measurement density $\rho_{D}^{-1}$ enters the minus input. The above structure is divided into two parts. The compandor in the upper part is:

(43) $$\begin{array}{c} f_{c}\left(x\right)=\left\{\begin{array}{ccc} -\rho_{D}^{-1}\left(1-e^{-\rho_{D}x}\right) & : & x\geq 0\\ \rho_{D}^{-1}\left(1-e^{-\rho_{D}x}\right) & : & x<0 \end{array}\right. \end{array}$$

The role of the above function compensates for the difference between the two inputs. That is, the inverse value of the estimated clutter measurement density should not be much different from the inverse value of the desired clutter measurement density. The output from the compandor comes out in square form, multiplied by $\beta *\delta_{k}$. β and $\delta_{k}$ are proportional constants and dither signals, respectively.

(44) $$\begin{array}{c} \beta =\rho_{D}^{2}\tau_{0} \end{array}$$

(45) $$\begin{array}{c} \delta_{k}=A_{Max}d^{k}{\left(-1\right)}^{k} \end{array}$$

In this structure, the input is the inverse of the clutter measurement density and the output is the detection threshold value. The role of β is to make the input equal to the dimension of the output. $\delta_{k}$ is a dither signal and acts as a stimulus to obtain the desired clutter measurement density. While the dither signal used in the existing extremum seeking control is a sine wave, the dither signal in the proposed extremum seeking control is set as a square wave [25]. $A_{Max}$ is set to $0.1\tau_{0}$, and $\tau_{0}$ is the initial detection threshold. $d^{k}$ is the decay factor and is set as $d^{k}={\left(0.9\right)}^{k}$. The amplitude of the dither signal is set large to reduce the difference between the two inputs in the initial period of the simulation. Since the estimated clutter measurement density converges to the desired clutter measurement density over time, the amplitude of the dither signal decreases over time.

The sign block at the bottom of the two parts is:

(46) $$\begin{array}{c} f_{s}\left(x\right)=\left\{\begin{array}{ccc} -1 & : & x>0\\ 1 & : & x<0\\ 0 & : & x=0 \end{array}\right. \end{array}$$

The role of the sign block is to determine whether to increase or decrease the detection threshold at the next scan based on the inverse of the estimated clutter measurement density at the current scan. For example, if the inverse of the estimated clutter measurement density is greater than the inverse of the desired clutter measurement density, this means that the estimated clutter measurement density at the current scan is less than the desired clutter measurement density. Therefore, the clutter measurement density at the next scan must be increased. To increase the clutter measurement density, the number of measurements is greater than the current scan, and the detection threshold $\tau_{k+1}$ must be smaller at the next scan. Thus, the sign of the output is minus. The detection threshold in the next scan can be calculated by adding the dither signal to the value obtained by applying numerical integration to the output values obtained from the two parts.

**Figure 2:**
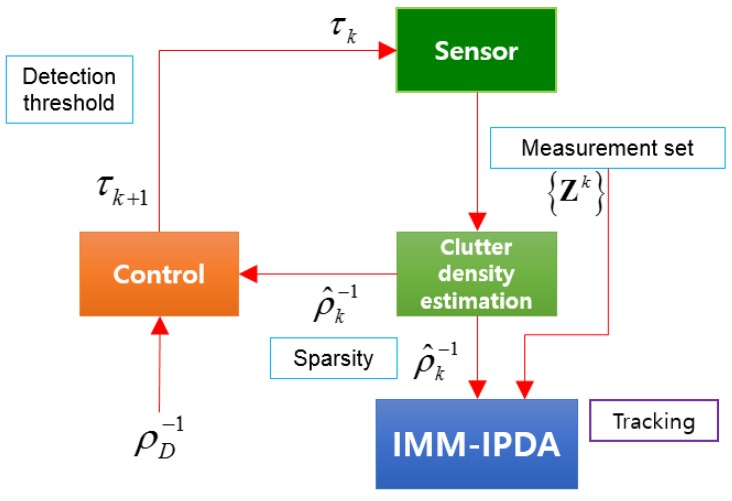
Structure of the target tracking system combined with the detection threshold adjustment. IMM-IPDA, interacting multiple model-integrated probabilistic data association.

Figure 2 shows the structure of the target tracking system combined with the detection threshold adjustment technique.

After extracting the information with a signal strength higher than the initial threshold, the clutter measurement density of the measurements is estimated. The estimated clutter measurement density is used to calculate the data association probability and the target existence probability in data association for the tracking filter. Then, the estimated clutter measurement density value is entered as the extremum seeking control input, and the detection threshold at the next scan is calculated.

## 4. Algorithm to Find the Desired Clutter Measurement Density

Before performing detection threshold control using extremum seeking control on-line for target tracking, a table for the desired clutter measurement densities should be prepared. The algorithm summarized in Algorithm 1 below is performed sequentially through specified off-line procedures in advance to find the desired clutter measurement densities for the tracking environment. The desired clutter measurement density is obtained as a function of $SNR_{dB}$. The table is used to find the desired clutter measurement density that matches the current tracking environment for the purpose of on-line extremum seeking control application. The clutter distribution for Algorithm 1 is assumed to be Chi-squared.

Table 1 summarizes the notations in Algorithm 1. First, a test is carried out by gradually reducing $P_{D}$ for a predetermined $SNR_{dB}$. Then, the probability of false alarm and ρ are calculated from the determined $P_{D}$ and $SNR_{dB}$. In this case, $P_{D}$ should be changed until a clutter measurement density that satisfies the $\rho_{Max}$ and $\rho_{Min}$ conditions is obtained in order to exclude the case where ρ is too high or too low. When this condition is satisfied, the IMM-IPDA is performed on the Monte Carlo simulation with a determined $P_{D}$ and ρ, varying the $T_{c}$ until the rate of CFT is approximately 0.005. When the rate of CFT is approximately 0.005, it saves the number of confirmed true tracks. Repeatedly performing the above steps results in different $nCTT(P_{D},\rho )$ depending on $P_{D}$ and ρ. The saved $nCTT(P_{D},\rho )$ values are compared, and a ρ value that satisfies $\rho_{D}$ = argmax${}_{P_{D}}\left\{nCTT\left(P_{D},\rho \right)\right\}$ is obtained as the desired clutter measurement density. This means that the number of confirmed true tracks is highest when applying the desired clutter measurement density. In other words, convergence of the estimated clutter measurement density to the desired clutter measurement density through detection threshold control can maximize the target tracking performance. The desired clutter measurement density obtained through this algorithm is used as the input value of the proposed extremum seeking control.

**Algorithm 1** Find the desired clutter measurement density.
1:Select $SNR_{dB}$
2:
**for** $P_{D}=0.99:-0.01:P_{D,Min}$ **do**
3: $P_{fa}=P_{D}^{1+SNR}$ where, $SNR=10^{\frac{1}{10}SNR_{dB}}$
4: $\rho =P_{fa}/V_{rc}$ where, $V_{rc}=\Delta x\Delta y$
5: $\Delta x=2\sqrt{3}\sigma_{x},\Delta y=2\sqrt{3}\sigma_{y}$
6: **if** $\rho >\rho_{Min}$ and $\rho <\rho_{Max}$ **then**7:  **for** $T_{c}=0.99:-0.01:T_{c,Min}$ **do**8:   **for** m=1:+1: the number of Monte Carlo **do**9:    IMM-IPDA($P_{D},\rho$) 10:   **end for**11:   **if** rate of CFT≈0.005 **then**12:    Record $nCTT(P_{D},\rho )$
13:   **end if**14:  **end for**15: **end if**16: $\rho_{D}$ = argmax${}_{P_{D}}\left\{nCTT\left(P_{D},\rho \right)\right\}$
17:
**end for**



## 5. Simulation

Two scenarios, where the probability distribution function (pdf) of the clutter signal strength was uncertain, were conducted to validate the effectiveness of the proposed method. In each scenario, both the fixed detection threshold commonly used in practice and the proposed adaptive detection threshold were simulated for comparative analysis.

In the first scenario, two constants λ were designated to model different pdfs of the clutter signal strengths to test the robustness under different environmental conditions. The IPDA algorithm was used for tracking the non-maneuvering target. Based on the analysis of the simulation results, a more complex scenario of maneuvering target tracking in a heterogeneous clutter environment was devised. In this scenario, the clutter distribution varied at different subareas in the surveillance region. Specifically, the surveillance region was divided into two subareas where each subarea had a different λ, and the target movement included crossing the border of the subareas. The target performed the maneuver twice with unknown turn rates. Therefore, the IMM-IPDA algorithm was used for better tracking performance in this scenario.

### 5.1. Scenario 1

**Figure 3:**
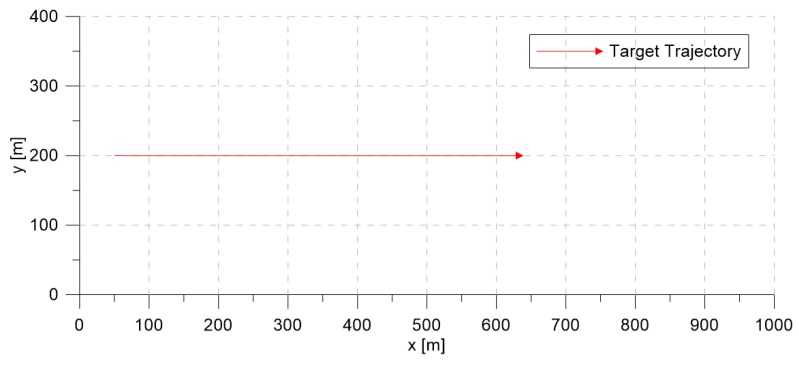
The non-maneuvering target trajectory in the surveillance region.

The target started from position [50m,200m${]}^{T}$ with a constant velocity 12 m/s, and the target trajectory is depicted in Figure 3. The standard deviations of the range and bearing measurements were $\sigma_{r}=5$ m and $\sigma_{\theta }=0.1^{{}^{\circ}}$, which are similar to the ones used in target tracking of [26], respectively. The active sonar at [−2000m,0m${]}^{T}$ measured the target information with sampling interval Δ=1 s, and the $SNR_{dB}$ was eight. The desired clutter measurement density obtained through the algorithm in Section 4 was $\rho_{D}=2.21\times 10^{-5}/$m${}^{2}$.

The performance index of the target tracking algorithm was performed through false track discrimination. The target existence probability, which was calculated using the likelihood ratio in data association, was used for the evaluation. A track with a target existence probability higher than a predetermined confirmation threshold value was defined as a confirmation track, and the FTD process was performed for each of the confirmed tracks during every scan. The FTD process played a role in distinguishing among these confirmed tracks whether the actual target was being tracked or the clutter was being tracked. If it satisfied the following equation, it was classified as a confirmed false track that was tracking the clutter:

(47) $$\begin{array}{c} {\left(x_{k}-{\hat{x}}_{k|k}\right)}^{T}{\left(P_{0|0}\right)}^{-1}\left(x_{k}-{\hat{x}}_{k|k}\right)>\tau_{False}. \end{array}$$

Here, $x_{k}$ and $P_{0|0}$ respectively represent the state vector of the actual target and the initial error covariance matrix when the track was initialized. The confirmed tracks were classified as confirmed true tracks, which were confirmed by actual targets when the following equation was satisfied:

(48) $$\begin{array}{c} {\left(x_{k}-{\hat{x}}_{k|k}\right)}^{T}{\left(P_{0|0}\right)}^{-1}\left(x_{k}-{\hat{x}}_{k|k}\right)<\tau_{True} \end{array}$$

In this simulation, $\tau_{False}$ and $\tau_{True}$ were set to 40 and 20, respectively. We used the CTTR (confirmed true track rate) and position RMSE (root mean squared error) as the performance index of the tracking filter. The CTTR represents the ratio of CTT per scan to Monte Carlo simulation. The definition for CTTR is $CTTR=\frac{nCTT}{M}$, where *M* and nCTT are defined as the total number of Monte Carlo simulations and the number of CTTs during a Monte Carlo simulation, respectively. The position RMSE is the calculated distance RMSE of the actual target and estimate for CTT. The formula for RMSE is expressed as:

(49) $$\begin{array}{c} RMSE=\sqrt{\frac{1}{M}\sum_{i=1}^{M}\left({\tilde{x}}^{2}+{\tilde{y}}^{2}\right)} \end{array}$$

where $\tilde{x}$ and $\tilde{y}$ are denoted by the difference between the true target and the estimate of CTT for *x* and *y*, respectively.

To analyze whether the estimated clutter measurement density closely follows the desired clutter measurement density when adjusting the detection threshold through extremum seeking control, the value of λ in the clutter signal strength probability distribution $P_{s}\left(s\right)=\lambda e^{-\lambda s}$ was varied as 1.2 and 0.8. Each simulation run had 50 scans, and we performed 500 Monte Carlo simulations.

**Figure 4:**
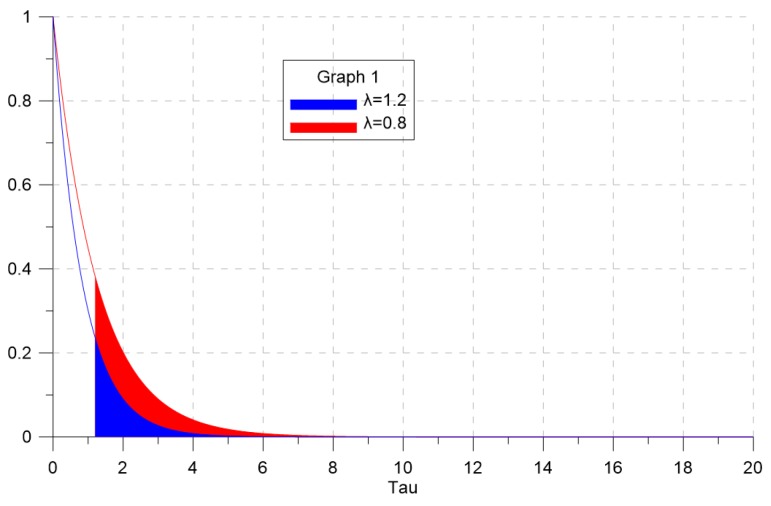
The probability of false alarm according to λ.

Figure 4 shows the clutter signal strength probability distribution and the probability of false alarm according to the value of λ. The blue line and the red line represent the probability distribution for λ=1.2 and λ=0.8, respectively. The smaller the value of λ, the greater the probability of false alarm. In other words, the smaller the value of λ, the greater the probability that the clutter signal strength will be higher near large tail of the pdf for signal strength. This means that the probability of clutter having a signal strength higher than the detection threshold increased.

#### 5.1.1. Results for λ=1.2

**Figure 5:**
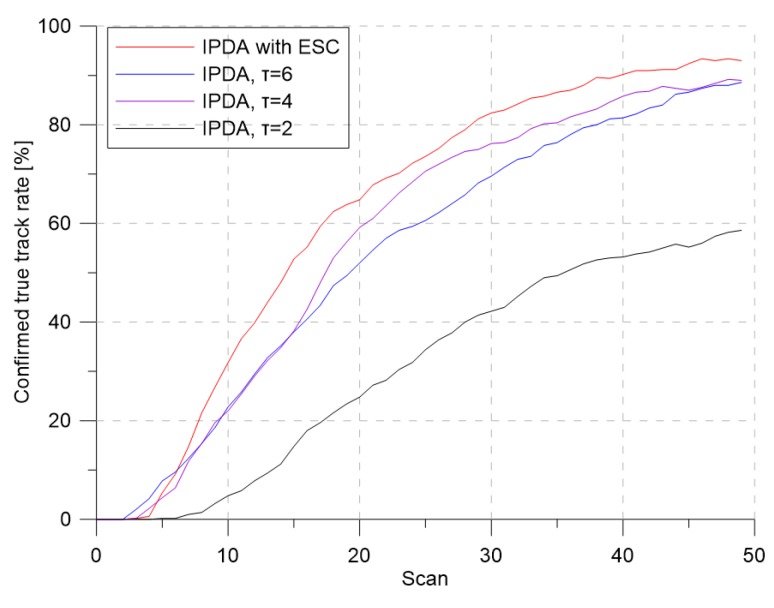
Confirmed true track rate for λ=1.2.

**Figure 6:**
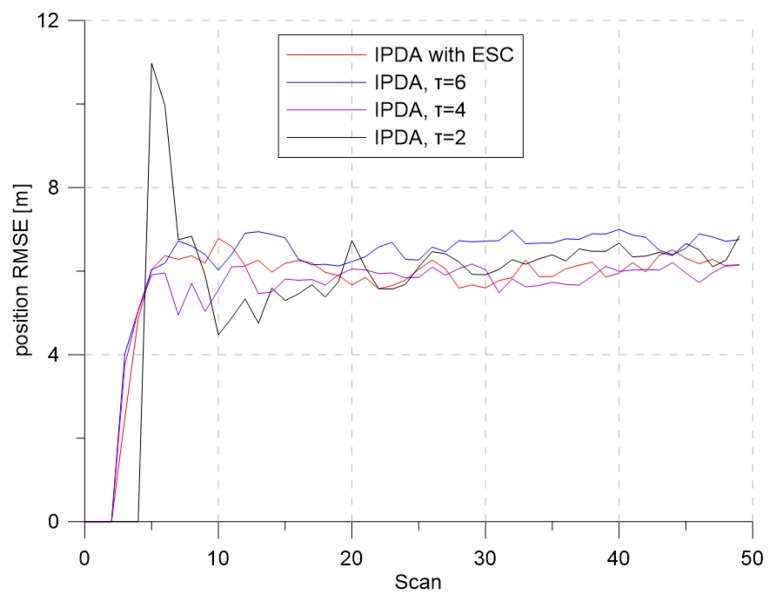
Position RMSE for λ=1.2.

Figure 5 and Figure 6 show the CTTR and position RMSE for Monte Carlo simulations, respectively. In the figure, the red line is the tracking result of the IPDA with the proposed detection threshold control with extremum seeking control, and the blue, purple, and black lines are the results when the detection threshold was fixed at 6, 4, and 2, respectively. In all four cases, the CTTRs did not reach 100 percent, but the CTTR result for IPDA with the extremum seeking control was seen to be the best. When the detection threshold was two, the clutter measurement density was 0.000302, and the detection probability of the target was 0.72. Although the clutter measurement density did not differ much from the desired clutter measurement density, the target tracking performance was somewhat different. This means that a small change in the clutter measurement density can have a significant impact on the target tracking performance. When the detection threshold was six, the clutter measurement density was 0.000002, and the detection probability of the target was 0.373. The clutter measurement density was much smaller than the desired clutter measurement density. Although the number of generated clutters was small due to the small clutter measurement density, the detection probability of the target also decreased proportionally; thus, the target tracking performance decreased because the frequency of track updating (which uses the target measurement) was low.

**Figure 7:**
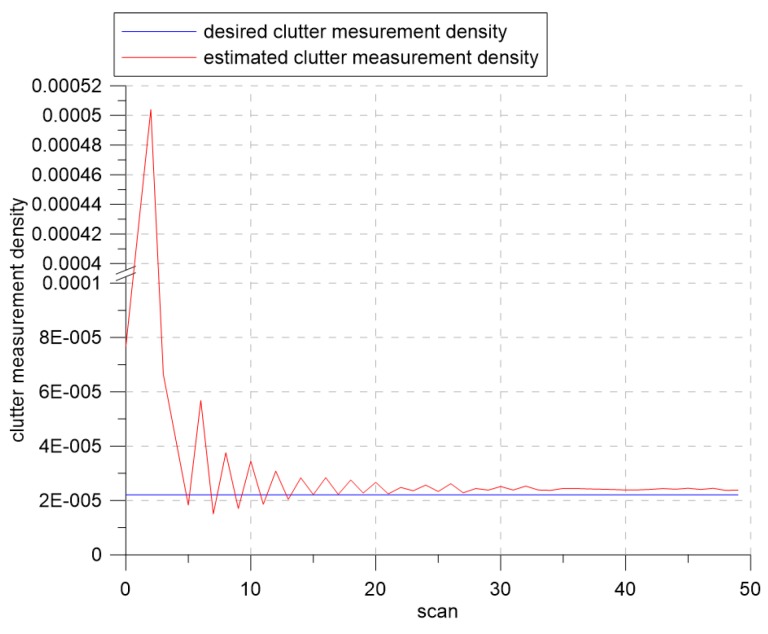
The desired clutter measurement density and estimated clutter measurement density for λ=1.2.

**Figure 8:**
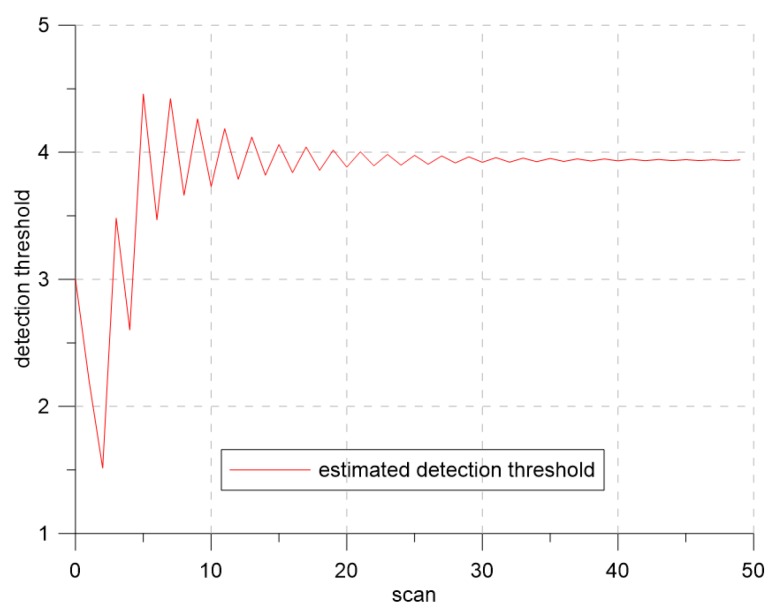
The detection threshold for λ=1.2.

Figure 7 and Figure 8 show the changes of the estimated clutter measurement density and the detection threshold, respectively, when using extremum seeking control. In Figure 7, the red and blue lines indicate the estimated clutter measurement density and the desired clutter measurement density, respectively. At the beginning of the simulation, the estimated clutter measurement density was much different from the desired clutter measurement density, but converged to the desired clutter measurement density value after Scan 25. In Figure 8, the detection threshold starts from the initial value of three and converges to the value of 3.94. Thus, this value is a detection threshold for which the estimated clutter measurement density converges to the desired clutter measurement density.

#### 5.1.2. Result for λ=0.8

**Figure 9:**
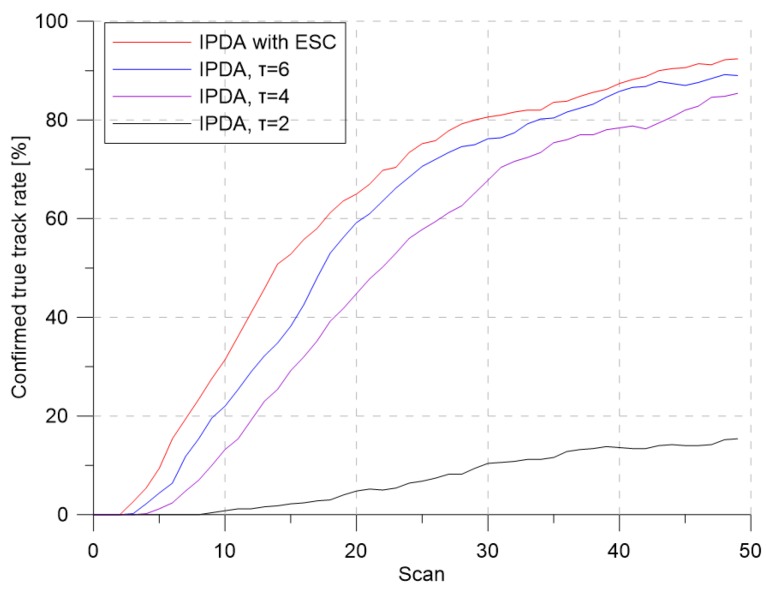
Confirmed true track rate for λ=0.8.

**Figure 10:**
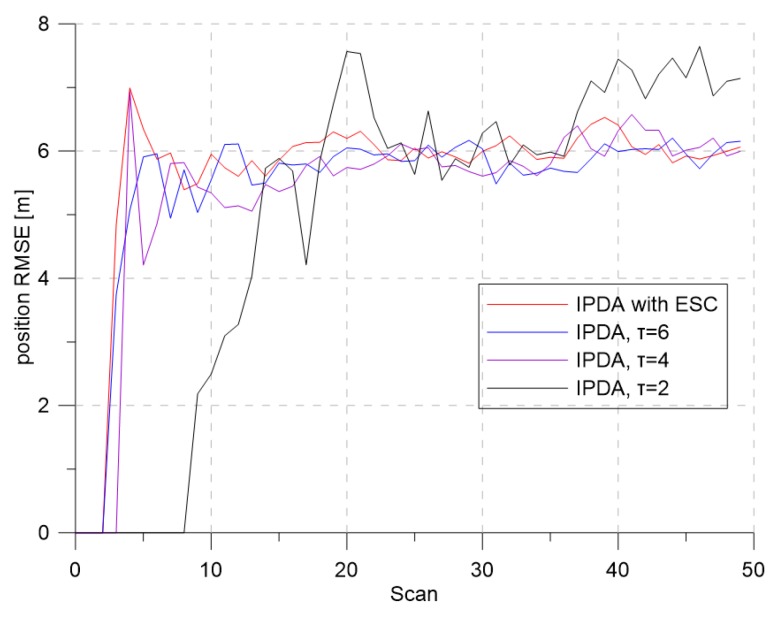
Range RMSE for λ=0.8.

Figure 9 and Figure 10 show the CTTR and position RMSE over time, respectively. When the detection threshold was two, the clutter measurement density was 0.000673, and the detection probability of the target was 0.803. In the above figures, it is seen that target tracking was not performed properly because the probability of false alarm was very high. The performance of the tracking filter with extremum seeking control was seen to be better even if the value of λ varied.

**Figure 11:**
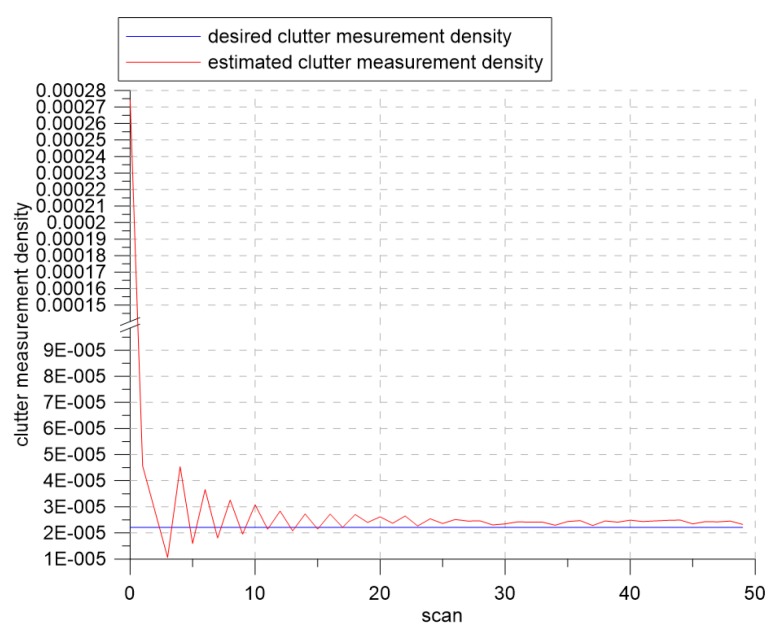
The desired clutter measurement density and estimated clutter measurement density for λ=0.8.

**Figure 12:**
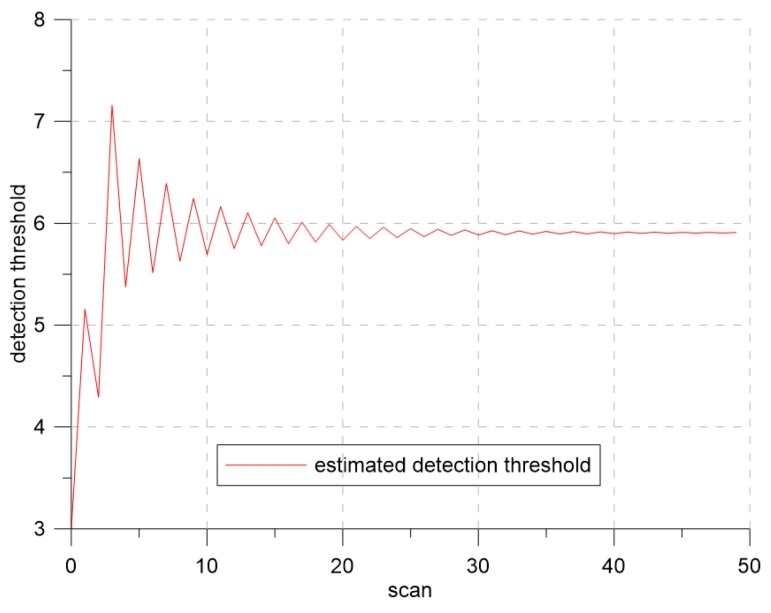
The detection threshold for λ=0.8.

Figure 11 shows that the variation of the estimated clutter measurement density at the beginning of the simulation was larger than that of Figure 7. As the signal strength distribution of clutter developed a large tail, the variation of the estimated clutter measurement density increased. However, we can see that estimated clutter measurement density converged to the desired clutter measurement density, similar to the results in the previous section. In Figure 12, the detection threshold started at three and converged to a value of 5.9. This shows that when λ was 0.8, the estimated clutter measurement density converged to the desired clutter measurement density when the detection threshold was 5.9.

To analyze the effect of the initial detection thresholds on the tracking performance, the simulation with varying the initial detection thresholds was performed. The initial detection thresholds were selected as $\tau_{0}$ = 2, 3, 4, and 5.

**Figure 13:**
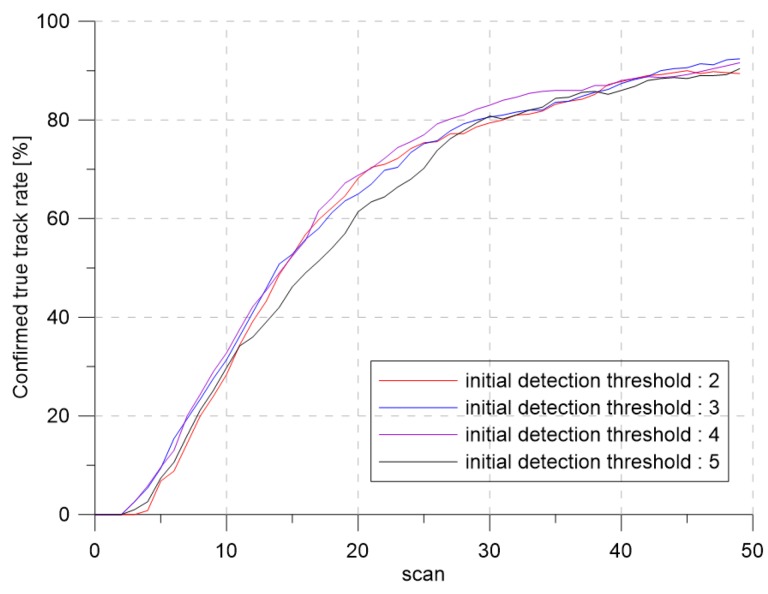
The confirmed true track rate according to each initial detection threshold.

**Figure 14:**
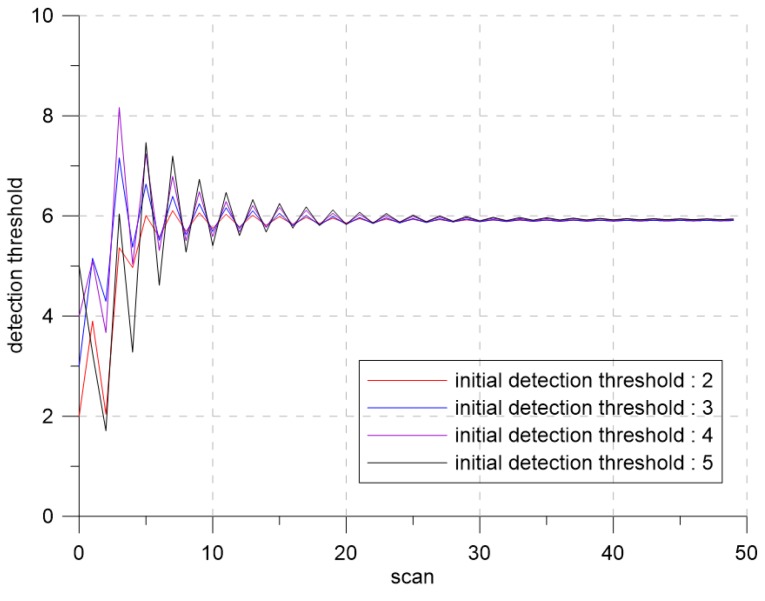
The detection threshold according to each initial detection threshold.

Figure 13 and Figure 14 show the confirmed true track rate and the detection thresholds over time for each case, respectively. Even though the initial detection threshold changed, the detection threshold converged to the same detection threshold, which produced the desired clutter measurement density with slight tracking performance difference.

### 5.2. Scenario 2

Scenario 2 was designed for checking the robustness of the proposed extremum seeking control algorithm by introducing a heterogeneous clutter environment (two regions with different λ), which was unexpected before the on-line application. Therefore, one $\rho_{D}$ was applied in both regions traveled by the target. The parameter settings were the same as those of Scenario 1. During the simulation run, the target had four maneuvering patterns, and Table 2 summarizes the target maneuvers over time.

**Figure 15:**
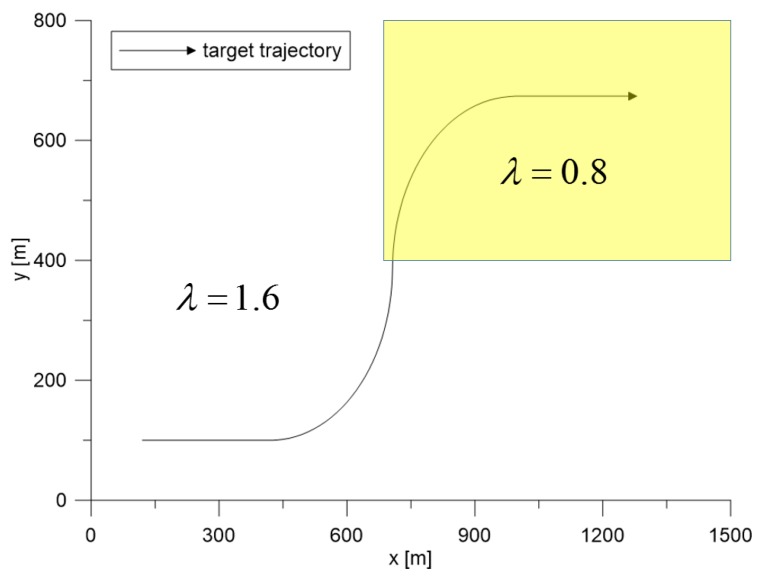
The maneuvering target trajectory in the surveillance region.

In Figure 15, the λ in the white region and the yellow region are 1.6 and 0.8, respectively. As discussed in Section 5.1, more clutter measurements were generated in the yellow region when the same detection threshold was used. The target entered the yellow region from Scan 50. The extremum seeking control adjusted the detection threshold based on the position of the track with the highest target existence probability among the updated tracks through IMM-IPDA. The desired clutter measurement density was $\rho_{D}=7.52\times 10^{-5}/$m${}^{2}$. The total number of Monte Carlo runs was 500, and each run consisted of 100 scans.

**Figure 16:**
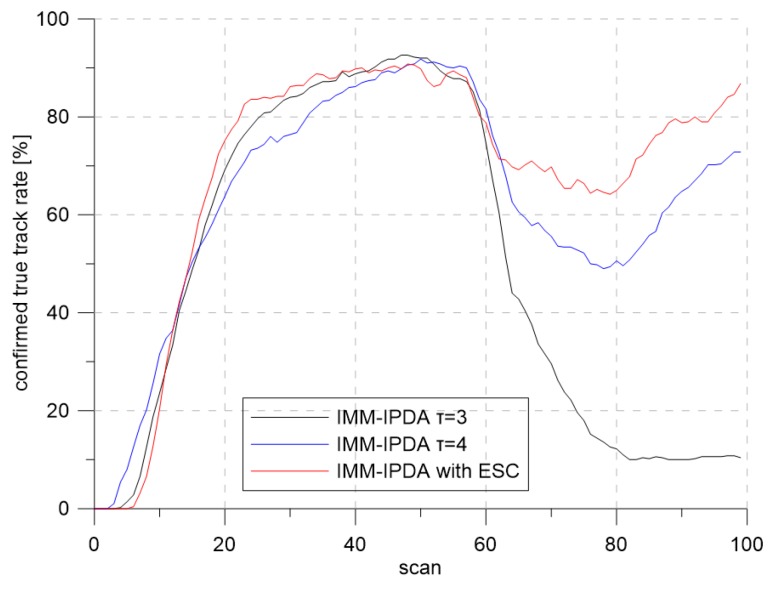
Confirmed true track rate for Scenario 2.

**Figure 17:**
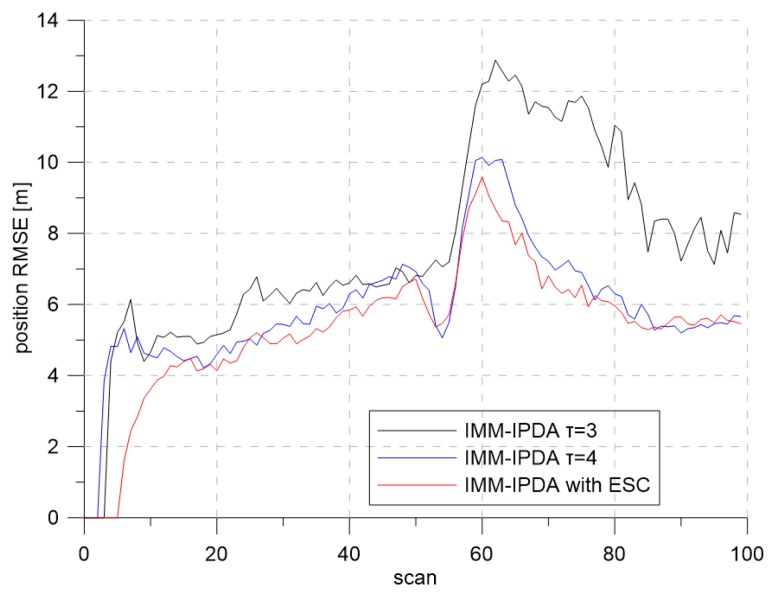
Position RMSE for Scenario 2.

**Figure 18:**
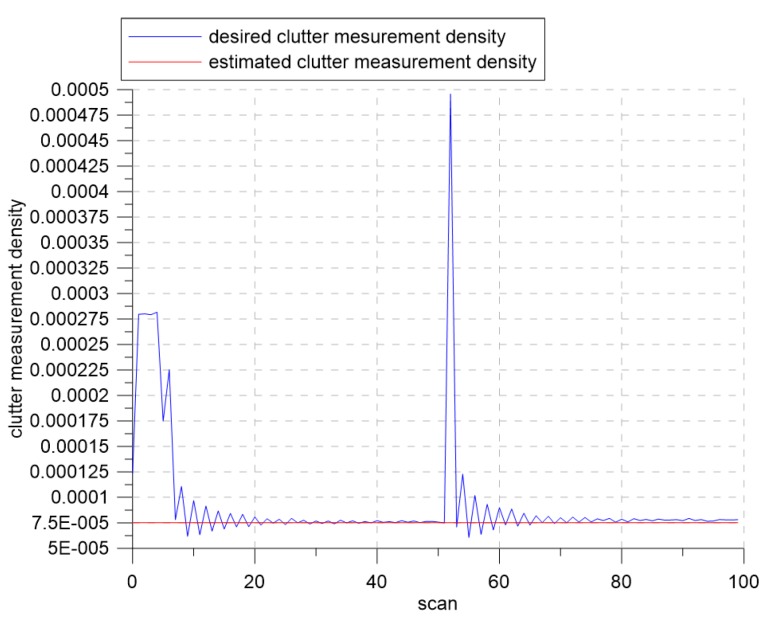
The desired clutter measurement density and estimated clutter measurement density for Scenario 2.

**Figure 19:**
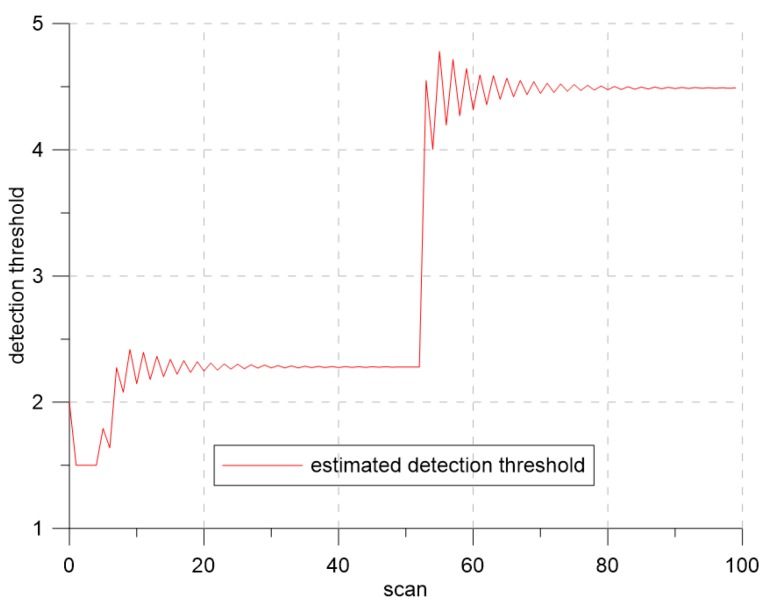
The detection threshold for Scenario 2.

Figure 16 and Figure 17 show the results of the CTTR and position RMSE over time in Scenario 2. The red line shows the tracking results of IMM-IPDA with the proposed detection threshold control method, and the black and the blue lines indicate the tracking results of IMM-IPDA with a detection threshold of three and four, respectively. When the detection threshold was fixed at three, the tracking performance was better than that of the case where the detection threshold was fixed to four in the white region, but the tracking performance was degraded in the yellow region. The estimated clutter measurement density when the threshold was fixed at three in the white area was closer to the desired clutter measurement density than the estimated clutter measurement density when the detection threshold was fixed at four. However, in the yellow region, the difference between the newly-estimated clutter measurement density and the desired clutter measurement density was further increased, and the tracking performance worsened. Figure 18 and Figure 19 show that the estimated clutter measurement density converged to the desire clutter measurement density by adjusting the detection threshold through the extremum seeking control even if the target moved to the region where λ changed.

## 6. Conclusions

In this paper, we proposed a detection threshold control method using extremum seeking control, considering the nonlinearity characteristics of the detection process, as well as an environment in which the probability density function of the signal strength for clutter was unknown. Since there was a trade-off between the detection probability of the target and the clutter measurement density, it is important to determine an appropriate detection threshold with optimal target tracking performance. To achieve optimal target tracking performance, the estimated clutter measurement density must converge to the desired clutter measurement. We conclude that the use of the IPDA algorithm with the proposed extremum seeking control was effective in improving the tracking performance in surveillance regions with a homogeneous clutter distribution, as the unknown clutter measurement density can be adjusted to the desired clutter measurement density by the proposed extremum seeking control algorithm. For maneuvering target tracking in heterogeneous clutter environments with unknown clutter distributions, IMM-IPDA with the proposed extremum seeking control produced better performance than the conventional methods with constant thresholds. This flexible feature contributes to its applicability for practical implementation. 

## Figures and Tables

**Table 1:** Notation.

Notation	Explanation
$SNR_{dB}$	signal-to-noise ratio in dB
$P_{D,Min}$	the detection probability of the target minimum limit
$\rho_{Max}$	clutter measurement density maximum limit
$\rho_{Min}$	clutter measurement density minimum limit
$V_{rc}$	resolution cell volume
$T_{c}$	confirmed threshold
$T_{c,Min}$	confirmed threshold minimum limit
rate of CFT	confirmed false track (CFT) scan/(total scan) × (total Monte Carlo)
$nCTT(P_{D},\rho )$	the total number of confirmed true tracks when set to $P_{D}$ and ρ
$\rho_{D}$	the desired clutter measurement density

**Table 2:** Target maneuvering patterns over time. CV, constant velocity; CCW, counterclockwise; CW, clockwise; CTR, constant turn rate.

Time (s)	Maneuvering Pattern	Turn Rate (${}^{{}^{\circ}}$/s)
1∼20	CV model	x
21∼50	CCW-CTR model	3
51∼80	CW-CTR model	3
81∼100	CV model	x
